# Supplementary figures and images for: Free energy landscape of RNA binding dynamics in start codon recognition by eukaryotic ribosomal pre-initiation complex
Source: PLoS Comput Biol. 2021 Jun 14;17(6):e1009068. doi: 10.1371/journal.pcbi.1009068 (PMC8224888; doi:10.1371/journal.pcbi.1009068)

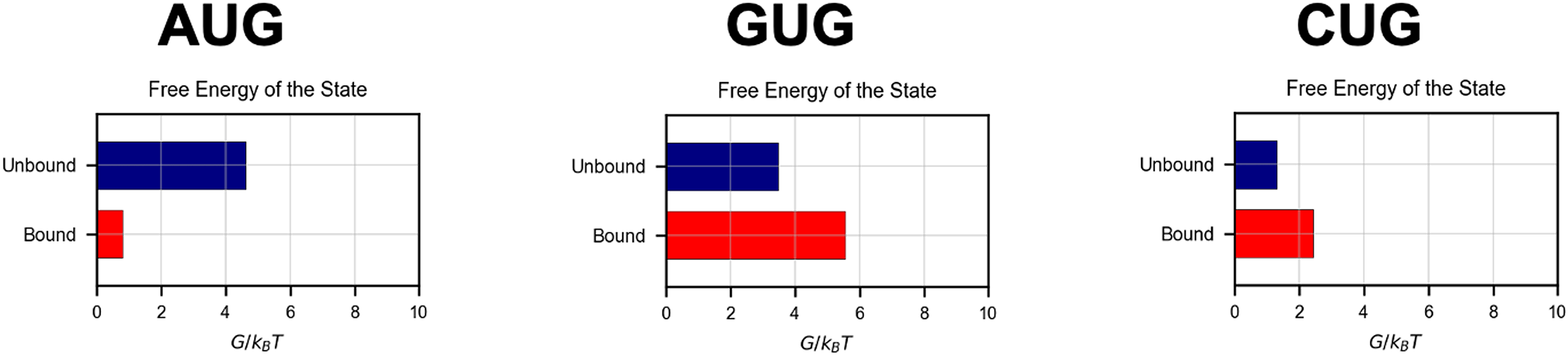

Supplement: S1 Fig — Red and blue bars correspond to Gbound and Gunbound (Eq 3), respectively (see the schematics in Fig 2). The scores were obtained from P(d1, d2, d3) averaged over five simulation trials for each model. (TIF) [file pcbi.1009068.s001.tif]

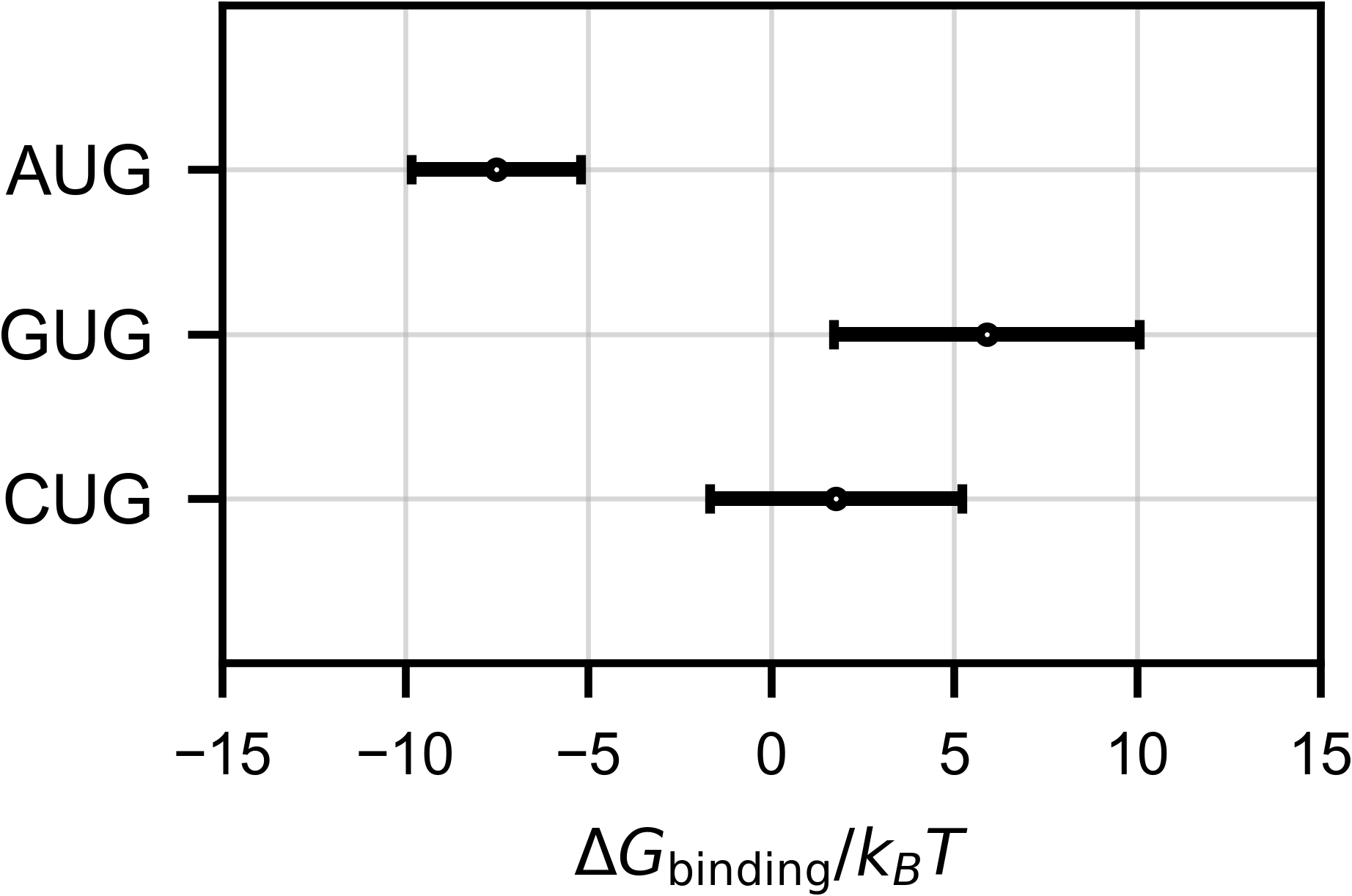

Supplement: S2 Fig — Error bars show the mean ± S.E.M. of ΔGbinding obtained from each simulation trajectory. Note that the mean ΔGbinding here is different from the ΔGbinding score in Fig 4, for which P(d1, d2, d3), not G, was averaged over simulation trials; this SI figure is presented only for reference and Fig 4 is physically relevant. (TIF) [file pcbi.1009068.s002.tif]
