# Supplementary material for: Free energy landscape of RNA binding dynamics in start codon recognition by eukaryotic ribosomal pre-initiation complex
Source: PLoS Comput Biol. 2021 Jun 14;17(6):e1009068. doi: 10.1371/journal.pcbi.1009068 (PMC8224888; doi:10.1371/journal.pcbi.1009068)
Supplement: Attachment — Submitted filename: cover.pdf [file pcbi.1009068.s003.pdf]

Dear Dr. Shakhnovich:

Thank you very much for the kind and timely review of our manuscript. We are glad to see that both the expert reviewers appreciated the novel computational approach to study start codon selection and concur with recommending a minor revision.

Please find attached the revised version of our manuscript, based on this review. Below, we address the reviewers' comments point-by-point. Our responses are written in boldface.

Thank-you again. We hope that the manuscript is now acceptable for publication in *PLoS Computational Biology*.

Best wishes,

Katsura Asano, Ph D

Professor, Kansas State University, USA

and

Yuichi Togashi, Ph D

Professor, Ritsumeikan University, Japan

Point-to-point response

Reviewer #1: "In this work, Kameda et al. performed adaptive biasing force molecular dynamics simulation to understand to free energy profiles for mRNA (codon) and the tRNA (anticodon) pairing. They further inferred the transition dynamics based on the free energy landscapes and explored atomic-level details of the start codon and near-cognate codon and their corresponding anticodon. Overall, this work enhanced the understanding of the mechanism of start codon and near-cognate codon in initiation of translation."

Thank you for acknowledging that the work enhanced the understanding of the mechanism of start codon selection in eukaryotic initiation.

"I only have several comments:

1. In Figure 3, it seems the curves are fluctuating instead of being more smoothed. Is this the nature of the simulation technique? Especially for CUG, it seems the curve keeps

going up and drops sharply close to the end. Also is that possible to have error bars on these curves?”

Our short answer for the first question is yes, and the second question, no.

More specifically, $L(\tau, \tau - \Delta\tau)$ (defined in eq. (5) and shown in Fig. 3) was used to evaluate the convergence of the (marginal) probability distribution sampled until τ, and L itself represents the fluctuations in the probability distribution. If the free energy landscape is strongly ragged (as shown in the case of CUG), the distribution may converge step by step, showing up-and-down in L. It is technically possible to calculate $L(\tau, \tau - \Delta\tau)$ for individual simulation trials and show error bars, however the meaning will become different.

Note that, unlike usual (unbiased) molecular dynamics simulations, the time τ here does not correspond to real timescales of the conformational changes, and rather represents the extent of sampling by the ABF method.

“2. The authors mentioned the free energy scores of bound and unbound states were averaged from five simulation trials. But I don't see any error/uncertainty estimation in Figure S1 and 4.”

It is possible to obtain the probability distribution P and calculate the Gibbs free energy G for each simulation trial. Then, assuming each trial as a sample, we can estimate the error in G. Indeed, we did so and confirmed the significance of $\Delta G_{\mathrm{binding}}$ by Welch’s *t*-test (presented in Results section in the original version of the text). In the revised manuscript, we added the result with error bars in Supporting Information Fig. S2.

However, due to nonlinearity of G in terms of P, the average of $\Delta G_{\mathrm{binding}}$ obtained above is different from that in Fig. 4, for which P (not G) is averaged over the simulation trials. From the physical point of view, the occurrence of each state (d_1, d_2, d_3) should be accumulated throughout the sampling, to obtain $G(d_1, d_2, d_3)$; hence, Figs. 4 and S1 (but not Fig. S2) are physically relevant. To avoid confusion, we kept Figs. 4 and S1 unchanged, and presented Fig. S2 for reference.

3. There are multiple panels in Figure 5. It would be better if they are all labeled (e.g., with letters) so that the authors could be more specific when they refer to these panels in the main text.

Thank you for this suggestion. Every panel of Figure 5 was labeled from (a) to (i), which was included in the parentheses referring to Figure 5 in the Results text.

“Reviewer #2: In this paper, Kameda et al report the free energy landscape of the interaction of the AUG start codon along with two non-cognate start codons (GUG and CUG) with the corresponding anti-codon on the ribosome in the eukaryotic pre-initiation complex using adaptive biasing force (ABF) molecular dynamics simulation. Contrary to earlier methods that have used free energy perturbation, ABF explicitly takes into account the conformations of the base pairs. The authors simulate several combinations of distances between base pairs in the three codon-anticodon pairs to compute their free energy landscape, which allows them to predict preferred transition pathways of between bound and unbound states. The authors demonstrate that between the two non-cognate start codons, GUG is less favorable than CUG, despite presence of the G-U wobble base pair in the former, and in the process are able to recapitulate the experimental observation that CUG is more frequently used than GUG. Additionally, the authors suggest a probable mechanism as to why the commonly observed GU wobble base pairing is less favorable in this case, predominantly due to steric hindrance caused by the beta-hairpin of eIF1. This work is important to mechanistically understand why certain codons are favored at the start position, and the methodology holds promise towards investigation of future RNA structural dynamics.”

We are excited to learn that this expert reviewer appreciates our work, mentioning that it lets us “*understand why certain codons are favored at the start position*”, and that “*the methodology holds promise towards investigation of future RNA structural dynamics*”.

“Comment: Based on the free landscape for GUG (Fig 6), the R1(GUG) structure (shown on top) seems more favored than where only the third base is paired (lower panel). However, in Fig 7, the structure that is shown for GUG has a different structure where the second and third base are paired. Unless I have missed something, this seems confusing.”

We apologize for the shortcomings of our description. Figure 6 indicates that the GUG scarcely forms triplet base-pairs simultaneously. One of the two plausible states represents base-pairing between GU and the corresponding bases in the anticodon, while the other is a base pairing between the 3rd G and C of the anticodon. In contrast, Figure 7 depicts the averaged structure in which every base of GUG and anticodon base-pairs is at a close proximity; which is indeed reconstructed from such very rare samples (note that the ABF method was adopted to efficiently sample rare states). Free energy for this structure is much higher than that for AUG structure, hence very low frequencies for GUG initiation. To resolve this confusion, we have added more explanation in the legends to the relevant figures (also related to minor point 2 of this reviewer).

“Minor:
1. In Fig 6, some base pairs are shown by blue dotted lines, while some are in red. It is not clear what this color code represents.”

We apologize again for our shortcomings. Blue indicates Watson-Crick, while red indicates wobble base-pairing. This is added to the legend of this figure.

"2. Figure legends in most cases would benefit from more details, about what different symbols and arrows suggest, and a small note about the significance of the figure, if possible. This would prevent a lot of back and forth reading."

As recommended, we added more details to Figure legends and small notes about the significance of the figure, where possible.

List of changes

- Fig. S2 has been added.
- Labels have been added to Fig. 5, and referred within the main text.
- Fig. 8 has been modified for clarity (*i. e.* same color-code used for RNAs).
- More details have been added to the caption of Figs. 6, 7, and 8.
- Definition of $P(d_1, d_2)$ has been corrected.

Changes in the text are indicated by red in the PDF version of the revised manuscript.
